# Dynasore suppresses cell proliferation, migration, and invasion and enhances the antitumor capacity of cisplatin via STAT3 pathway in osteosarcoma

**DOI:** 10.1038/s41419-019-1917-2

**Published:** 2019-09-18

**Authors:** Binlong Zhong, Deyao Shi, Fashuai Wu, Shangyu Wang, Hongzhi Hu, Cheng Cheng, Xiangcheng Qing, Xin Huang, Xueying Luo, Zhicai Zhang, Zengwu Shao

**Affiliations:** 1 Department of Orthopaedics, Union Hospital, Tongji Medical College, Huazhong University of Science and Technology, 1277 JieFang Avenue, Wuhan, 430022 China; 2 Tongji Medical College, Huazhong University of Science and Technology, Wuhan Mental Health Centre, Wuhan Hospital for Psychotherapy, Wuhan, China

**Keywords:** Bone cancer, Drug discovery, Drug development

## Abstract

Osteosarcoma (OS) is the most common malignant bone tumor. The prognosis of metastatic and recurrent OS patients still remains unsatisfactory. Cisplatin reveals undeniable anti-tumor effect while induces severe side effects that threatening patients’ health. Dynasore, a cell-permeable small molecule that inhibits dynamin activity, has been widely studied in endocytosis and phagocytosis. However, the anti-tumor effect of dynasore on OS has not yet been ascertained. In the present study, we suggested that dynasore inhibited cell proliferation, migration, invasion, and induced G0/G1 arrest of OS cells. Besides, dynasore repressed tumorigenesis of OS in xenograft mouse model. In addition, we demonstrated that dynasore improved the anti-tumor effect of cisplatin in vitro and in vivo without inducing nephrotoxicity and hepatotoxicity. Mechanistically, dynasore repressed the expression of CCND1, CDK4, *p*-Rb, and MMP-2. Furthermore, we found that dynasore exerts anti-tumor effects in OS partially via inhibiting STAT3 signaling pathway but not ERK-MAPK, PI3K-Akt or SAPK/JNK pathways. P38 MAPK pathway served as a negative regulatory mechanism in dynasore induced anti-OS effects. Taken together, our study indicated that dynasore does suppress cell proliferation, migration, and invasion via STAT3 signaling pathway, and enhances the antitumor capacity of cisplatin in OS. Our results suggest that dynasore is a novel candidate drug to inhibit the tumor growth of OS and enhance the anti-tumor effects of cisplatin.

## Introduction

Osteosarcoma (OS) remains the most common malignant bone tumor with a preference for the metaphysis of tubular long bones, especially in distal femur, proximal tibia and humerus, and most occurs in adolescents and teenagers. The incidence of OS is only 1.7–4.4 per million^1^, but with great invasive and metastatic capacity, the progression of OS squint towards disability and death, which causes substantial psychological and financial burdens. By following the treatments of neoadjuvant chemotherapy and surgical resection followed by adjuvant chemotherapy, the 5-year even-free survival rate reaches 60–70% in patients with localized, non-metastasis OS^2^. However, most patients present metastasis, usually in lung, when first diagnosed, and encounter poor prognosis with 5-year survival rate of 20–30% even they adhere to standard therapy strategies^3,4^. Even worse, the diverse side-effects limit the choices and usages of anti-tumor drugs in OS chemotherapy. As one of the crucial drugs in OS chemotherapy, cisplatin exerts a potent anti-OS activity, but at the same time, causes apparent side effects including nephrotoxicity, hepatotoxicity, ototoxicity, and myelosuppression^5,6^. Cisplatin induced nephrotoxicity is the most common side effect, which is confirmed to be dose-duration-frequency dependent^7^. Higher cumulative dose and higher doses per treatment of cisplatin will result in greater kidney injury irreversibly^8–10^. Thus, it is necessary to establish novel effective drugs with no or less side effects for OS chemotherapy.

Dynasore is a cell-permeable small molecule that non-competitively inhibits the GTPase activity of dynamin, which is a protein essential for cell adhesion, invasion, endocytosis, and phagocytosis^11^. Since identified by Macia in 2006, dynasore was widely utilized in the studies of endocytosis and macropinocytosis^11,12^. Recently, literatures have found that dynasore plays protective role in spinal injury^13^, Alzheimer disease^14^, and heart ischemia/reperfusion injury^15^. In addition, dynasore suppresses the pseudopodia formation and cell invasion by destabilizing F-actin^16,17^. Moreover, in the latest study, dynasore exhibited anti-cancer potential via inhibiting cell proliferation and migration while induced apoptosis and mitochondrial dysfunction in lung cancer cell^18,19^. However, the anti-tumor effect of dynasore on OS has not yet been ascertained.

In the present study, we demonstrated that dynasore inhibited cell proliferation, migration, invasion, and tumorigenesis of OS without inducing cell apoptosis. By combining cisplatin and dynasore, we found that dynasore enhanced the anti-OS effect of cisplatin in vitro and in vivo. Furthermore, ERK-MAPK, PI3K-Akt, SAPK/JNK, p38 MAPK, and JAK2-STAT3 pathways were assessed to identify the underlying mechanisms of the anti-proliferation effect of dynasore on OS.

## Materials and methods

### Cell lines and cell culture

All the OS cell lines (MNNG/HOS Cl#5, MG-63, and U2-OS) were purchased from CBTCCCAS (Cell Cank, Type Culture Collection, Chinese Academy of Sciences) (Shanghai, China) and identified by STR analysis. All the three cell lines were cultured in DEM/F12 medium, supplemented with 10% fetal bovine serum (FBS), and incubated in 37 °C, 5% CO_2_ incubator.

### Reagents

Dynasore was purchased from Target molecule Corp. (Targetmol, Shanghai, China) and dissolved with dimethylsulfoxide (DMSO, Sigma, USA) to a stock concentration of 100 mM. Cisplatin was bought from the National Institute for Food and Drug Control of China and dissolved with 0.9% normal saline solution (NS). To explore the inhibitory effects of dynasore and cisplatin, the cell viability of OS cells were detected in a serial concentration gradient (0, 10, 20, 50, 100 μM for dynasore, and 0, 5, 10, 20, 50 μM for cisplatin) for 24 h, 48 h, and 72 h. Unless otherwise specified, the rest in vitro experiments were performed on four groups treated with 0.05% DMSO (control group), 50 μM dynasore, 5 μM cisplatin, or 50 μM dynasore combined with 5 μM cisplatin (DC, combine group). To inhibit p38 MAPK signaling pathway, a specific inhibitor named SB239063 (MCE, China) was dissolved with DMSO and added 2 h before dynasore (50 μM) treatment for a final concentration of 10 μM. IL6 (50 ng/ml, PeproTech, USA) was administrated 1 h before dynasore (50 μM) to activate JAK2-STAT3 signaling.

### Cell proliferation assay

The OS cell viabilities were detected by cell count kit-8 (CCK-8, Dojindo Molecular Technologies, Japan) according to the instruction of the manufacture. In brief, 5 × 10^3^ OS cells were inoculated and cultured in a 96-well plate with 100 μl culture medium. After treatment with different reagents, the culture medium were replaced with 100 μl CCK-8 detection solution (90 μl fresh medium mixed with 10 μl CCK-8 solution). After incubation in cell incubator (37 °C, 5% CO_2_) for 2 h, the absorbance were valued via a microplate reader at 450 nm (Biotek, Winooski, VT, USA), and cell viability were calculated according to the instruction. To observe the proliferative cells intentionally, 5-ethynyl-20-deoxyuridine (EdU) incorporation assay was implemented using Cell-Light^TM^ EdU Apollo® 567 in vitro imaging kit (C10310-1, RiboBio, Guangzhou, China) according to the manufacture’s instruction. The EdU assays were performed by three independent experiments, and cell counting was implemented by two investigators independently using the ImageJ software (National Institutes of Health, USA).

### Cloning formation assay

After trypsinized into a single-cell suspension, OS cells (500 cells per well) were seeded and cultivated in a 6-well plate for 24 h. Then, the cells were treated with DMSO, dynasore, and cisplatin with/without dynasore for another 48 h. Culture medium was replaced into fresh DEM/F12 medium with 10% FBS after drugs treatment, and the medium was changed every 2 days. After 10 days of culture, the cells were fixed with 4% paraformaldehyde for 20 min and stained with 1% crystal violet for 30 min. Clones were photographed by camera, and the clones with more than 50 cells were counted using microscope by two investigators. All the cloning formation assays were repeated three times.

### Wound healing assay

4 × 10^5^ cells/well were inoculated into 6-well plate and maintained in 37 °C, 5% CO_2_ incubator for 24–48 h until cells reached 100% confluence. Then the cell monolayer was scratched to create a gap with 200 μl pipette tip followed by PBS washing. Finally, the culture medium was changed into serum free DEM/F12 medium to suppress cell proliferation. The gaps were observed and photographed at 0 h and 24 h after scratching, and the area of scratches were calculated by ImageJ software.

### Cell migration and invasion assays

The MNNG/HOS and MG-63 cells were treated with DMSO, dynasore, cisplatin, and dynasore combined with cisplatin for 48 h. Then cells were trypsinized and 4–8 × 10^4^ cells in 200 μl serum free DEM/F12 were sucked into upper chamber of transwell (8μm pore size, BD Biosciences, St Louis, USA) with or without pre-coated Matrigel (BD Biosciences, St Louis, USA) to evaluate cell invasion and migration capacity, respectively. 600 μl culture medium containing 20% FBS was added into the lower chamber to stimulate cell travelling. After 36 h culture at 37 °C, 5% CO_2_ incubator, transwell chambers were fixed using 4% paraformaldehyde, and then stained with 1% crystal violet. Cells laid on upper surface of transwell membrane were wiped using a cotton swab, while cells traveled to the lower surface of membrane were photographed under a microscope. The average transmitted cells were counted by two investigators in three random fields (100×).

### Flow cytometric analysis

After treated with DMSO, dynasore, cisplatin, and dynasore combined with cisplatin for 48 h, MNNG/HOS and MG-63 were harvested to perform flow cytometric analysis. For cell cycle distribution analysis, cells were washed by clod PBS and fixed with pre-cold 70% ethanol overnight in 4 °C. Then the cells were stained using Cell Cycle and Apoptosis Analysis Kit (Beyotime Biotehnology, China). In brief, cells were incubated in 37 °C incubator with propidium iodide (PI) working solution for 30 min shielded from light. The cell cycle distribution was assessed via Flow Cytometry Caliber instrument (BD Biosciences, USA). Cell apoptosis assessment was performed by PE Annexin V Apoptosis Detection Kit I (BD Biosciences, USA) according to the instruction of the manufacture. After stained with 7-Amino-Actinomycin (7-AAD) and PE Annexin V for 15 min at room temperature, the cell apoptosis was analyzed by Flow cytometer. The 7-AAD negative and PE Annexin V positive cells were considered as early apoptotic cells, while those with double positive were determined as in late apoptosis or dead.

### Western blot analysis

The total cell protein was extracted by RIPA lysis buffer containing cocktail protease inhibitor and phosphorylase inhibitor (Servicebio technology, China). Protein concentrations were determined by using BCA protein assay kit (Cwbiotech, China). Then, 20 μg proteins were separated via 12% SDS-PAGE and transferred into PVDF membrane (0.22 μm, Millipore, Massachusetts, USA). After blocked by 0.5% defatted milk, membranes were incubated with diluted specific primary antibody and HRP conjugated secondary antibody, successively. Primary antibodies used in the present study include anti-GAPDH (1:10000, 10494-1-AP, Proteintech), anti-human antibody CDK4 (1:1000, ab108357, Abcam), anti-human antibody CCND1 (1:1000, ab40754, Abcam), anti-human antibody *p*-Rb (1:500, sc-24585, Santa Cruz), anti-N-cadherin (1:1000, #1311, Cell Signaling Technology), anti-MMP-2 (1:1000, 10373-2-AP, Proteintech), anti-MMP-9 (1:1000, 10375-2-AP, Proteintech), anti-JAK2 (phospho Y1007 + Y1008) (1:1000, ab32101, Abcam), anti-JAK2 (1:2000, ab108596, Abcam), anti-STAT3 (phospho Y705) (1:1000, ab76315, Abcam), anti-STAT3 (1:1000, ab68153, Abcam), anti-p38 (phospho T180) (1:1000, ab178867, Abcam), anti-p38 (1:1000, ab170099, Abcam), anti-Akt(phosphor S473) (1:1000, 66444-1-Ig, Proteintech), anti-Akt (1:1000, 10176-2-AP, Proteintech), anti-ERK1/2 (phospho T202 + T204)(1:1000, ab214362, Abcam), anti-ERK1/2 (1:5000, ab184699, Abcam), anti-SAPK/JNK (1:1000, #9252, Cell Signaling Technology), anti-SAPK/JNK (phosphor Thr183/Tyr185) (1:1000, 81E11, Cell Signaling Technology). The secondary antibodies include HRP-conjugated affinipure goat anti-mouse IgG (1:5000, SA00001-1) and anti-rabbit IgG (1:5000, SA00001-2) both were purchased from Proteintech group, USA. The PVDF membranes were analyzed using ECL (Enhanced Chemiluminescence) Plus reagents and scanned with the BioSpectrum Imaging System (UVP, German).

### RNA isolation and quantitative real-time PCR

The total RNA of MNNG/HOS and MG-63 was isolated using TRIzol® Reagent (Life Technologies, USA) after treated with reagents described above. One μg of total RNA was reverse-transcribed using ReverTra Ace® qPCR RT kit (TOYOBO, Japan) according to the manufacturer’s instructions. Quantitative real-time PCR was performed in the CFX Connect™ Real-Time PCR Detection System (Bio-Rad, USA) by using TB Green^TM^ Premix Ex Taq^TM^ (TAKARA, Japan). The CT values were normalized to GAPDH expression, and mRNA expressions were calculated via 2^−ΔΔCt^ method as described in previous research^20^. The primers used in present study are listed in Table 1.

**Table 1:** Primers used in quantitative real-time PCR

Gene	Forward primer (5′ → 3′)	Reverse primer (5′ → 3′)
CDK4	TCAGCACAGTTCGTGAGGTG	GTCCATCAGCCGGACAACAT
CCND1	GCTGCGAAGTGGAAACCATC	CCTCCTTCTGCACACATTTGAA
STAT3	CAGCAGCTTGACACACGGTA	AAACACCAAAGTGGCATGTGA
GAPDH	AATCCCATCACCATCTTCCAG	GAGCCCCAGCCTTCTCCAT

### In vivo assay

The in vivo experiment of this study was accomplished with the approval of the ethical committee of Tongji Medical College, Huazhong University of Science and Technology, and followed the institutional guideline and ethical standard. To establish subcutaneous transplanted model of OS, 3 × 10^6^ MNNG/HOS cells were injected into the right axilla of each 5-week-old male nude mouse (BALB/c-nu, Beijing HFK Bioscience, China). The xenografts were measured by micrometer caliper, and the tumor volume was calculated based on length (a) and the width (b) by the following formula: tumor volume = ab^2^. When xenografts volume reached 80–100 mm^3^, nude mice were divided into four groups randomly and received the following interventions every day: (1) in control group, mice were peritumoral subcutaneously and intraperitoneally injected with 100 μl NS respectively; (2) in Dynasore group, 100 μl NS with or without dynasore (10 mg/kg) were peritumoral subcutaneous and intraperitoneal injected, respectively; (3) in Cisplatin group, 100 μl NS without or with cisplatin (2 mg/kg) were peritumoral subcutaneous and intraperitoneal injected, respectively; (4) in combination group(DC), 100 μl NS containing dynasore (10 mg/kg) and 100 μl NS supplemented with cisplatin (2 mg/kg) were injected peritumoral subcutaneously and intraperitoneally, respectively. To explore the role of p38 MAPK pathway in dynasore induced anti-proliferation effect of OS in vivo, another xenograft mouse model was performed as described above, and the cisplatin (2 mg/kg) treatment was replaced with 10 mg/kg SB239063^21^ (intraperitoneal injection). All the mice were sacrificed after intervened for one week, and xenografts, livers and kidneys were resected carefully and fixed in 4% paraformaldehyde for H&E staining and immunohistochemistry assay.

### Histopathology and immunohistochemistry

The samples were embedded in paraffin and sectioned to 4 μm thickness. The sample sections (tumors, livers, and kidneys) were dewaxed and stained with Hematoxylin and Eosin Staining Kit (Beyotime Biotechnology, China) according to the manufacture’s instruction. Additionally, to explore the protein expression in OS xenografts, the tumor sections were used to perform immunohistochemistry with Ki67 (1:200, ab16667, Abcam), STAT3 (1:100, ab68153, Abcam) or *p*-STAT3 (1:100, ab76315, Abcam) primary antibodies and a biotin-conjugated affinipure goat anti-rabbit IgG (1:500, SA00004-2, Proteintech). For negative controls, immunohistochemistry was carried out in the absence of primary antibodies.

### Immunofluorescence assay

2–4 × 10^4^/well MNNG/HOS and MG-63 cells were seeded on circle cover glass (φ = 20 mm) in 12-well plate, and treated with 0.05% DMSO or 50 μM dynasore for 48 h. Cells were then fixed with 4% paraformaldehyde at 4 °C for 15 min, and permeabilized with 1% triton X-100 for 20 min. After 1 h blocking in PBS containing with 10% FBS, cells were incubated in STAT3 (1:500, ab68153, Abcam) or p-STAT3 (1:500, ab76315, Abcam) primary antibodies at 4 °C overnight. Then, the cells were washed with PBST for three times (5 min each time), and incubated with Dylight 488 goat anti-rabbit IgG secondary antibodies (1:500, Abbkine, USA) for 2 h at room temperature. Cell nucleus was stained via Hoechst 33342 (Beyotime Biotechnology, China) for 5 min at room temperature.

### Statistical analysis

Results of the present study were expressed as mean ± SEM for at least three independent experiments and analyzed by using SPSS 21.0 software (IBM, Chicago, IL, USA) and GraphPad Prism 6. Two-tailed student’s *t*-test was used to examine significant difference between any two groups, while one-way ANOVA was performed to assess the statistical significance between three or more groups. *P* < 0.05 was considered as statistically significant.

## Results

### Dynasore inhibits cell proliferation of OS in vitro

**Fig. 1:**
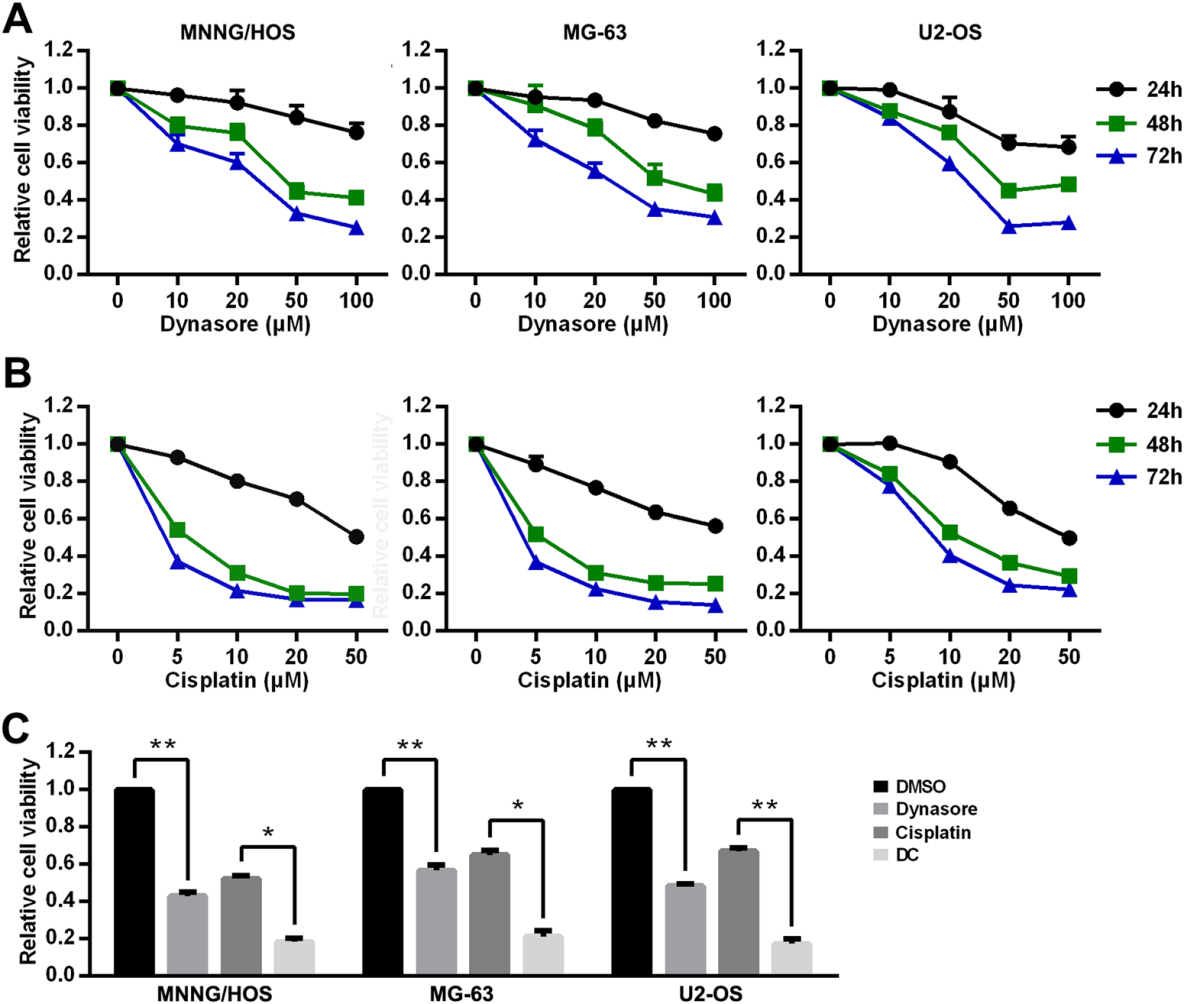
Inhibitory effect of dynasore and cisplatin on the cell viability of OS cells (MNNG/HOS, MG-63, and U2-OS cell lines). **a** Cell viabilities of OS cells after treated with different concentrations of dynasore for 24, 48, and 72 h. **b** Cell viabilities of OS cells after treated with different concentrations of cisplatin for 24, 48, and 72 h. **c** Cell viabilities of OS cells after treated with 50 μM dynasore and/or 5 μM cisplatin for 48 h. *p < 0.05 and **p < 0.01, vs. DMSO group. DC, 50 μM dynasore combined with 5 μM cisplatin

Human OS cell lines (MNNG/HOS, MG-63, and U2-OS) were treated with increasing concentrations of dynasore or cisplatin, and then the cell viability was assessed by CCK-8 kit at 24, 48, and 72 h. As shown in Fig. 1a, b, the cell abilities of MNNG/HOS, MG-63, and U2-OS were suppressed in a time- and concentration-dependent manner either treated with dynasore or cisplatin. To evaluate the synergistic antitumor effect of dynasore and cisplatin, cells were exposed to dynasore (50 μM) or/and cisplatin (5 μM) for 48 h. As expected, cell viabilities were significantly decreased in dynasore group and cisplatin group, while those in dynasore and cisplatin combination group (DC group) were further suppressed (Fig. 1c). Besides, the CDIs (Coefficients of Drug Interaction) of 50 μM dynasore and 5 μM cisplatin in MNNG/HOS, MG-63, and U2-OS cells were reached up to 0.80 ± 0.03, 0.58 ± 0.05, and 0.54 ± 0.05, which means dynasore promoted the antitumor capacity of cisplatin. Then, the effects of dynasore and cisplatin alone or in combination were analyzed via observing cell morphology. Unlike apoptosis-inducing feature of cisplatin, dynasore exhibited a significant inhibitory effect on cell density. In DC group, both anti-proliferation and apoptosis-inducing effects were observed (Supplemental Figs. 1–3).

**Fig. 2:**
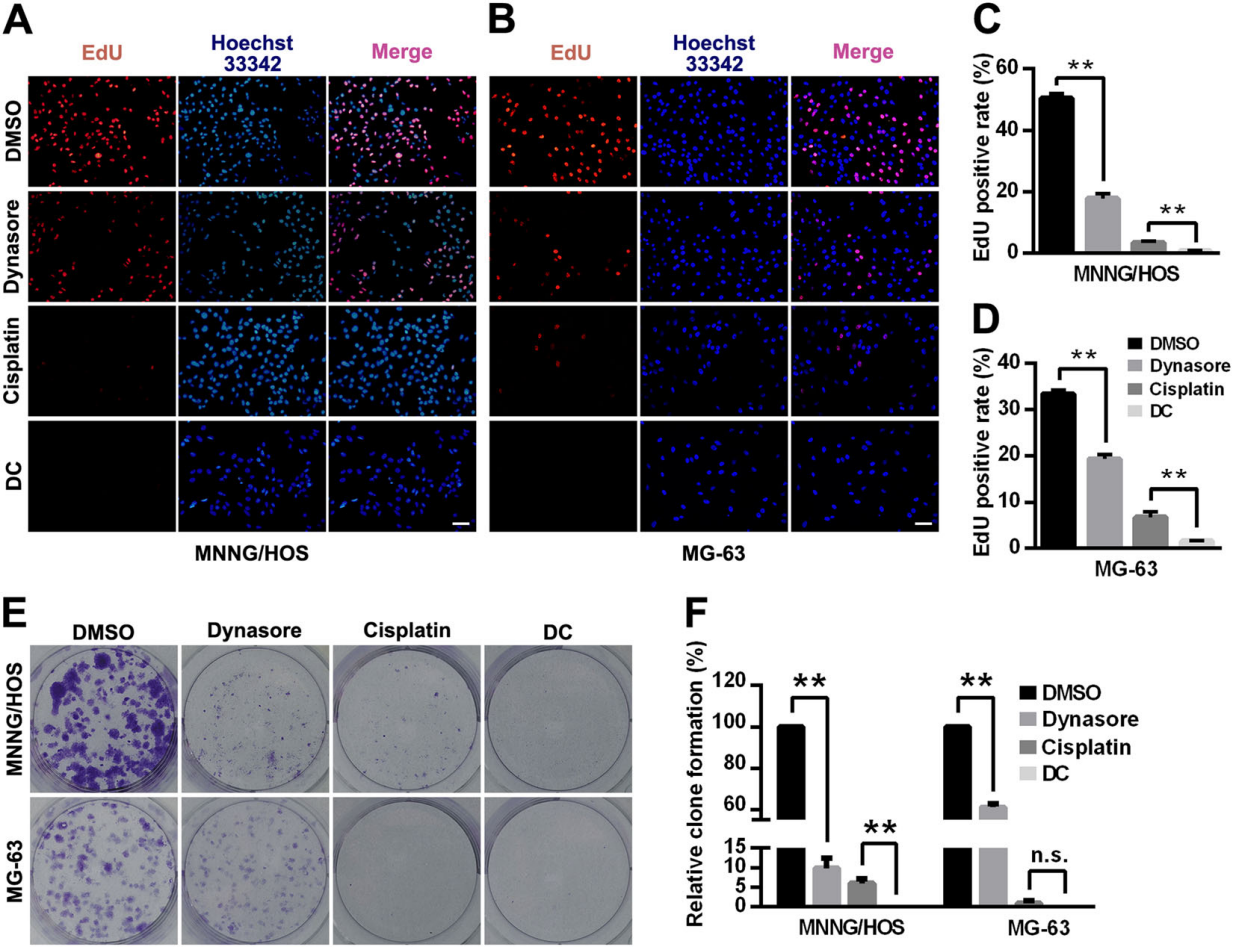
Inhibitory effect of dynasore and/or cisplatin on cell proliferation of OS cells. Cell proliferation was detected by EdU staining in MNNG/HOS (**a**) and MG-63 (**b**). EdU positive rates of MNNG/HOS (**c**) and MG-63 (**d**) were shown as histograms. **e** Cloning formation of MNNG/HOS and MG-63 after treated with 50 μM dynasore and/or 5 μM cisplatin for 48 h. **f** Histograms of relative cloning formation of OS cells. **p < 0.01 vs. DMSO group. n.s., not significant. Scale bar: 50 μm

To further estimate the anti-proliferation effect of dynasore on OS cells, EdU staining was performed in MNNG/HOS and MG-63 cells. As depicted in Fig. 2a–d, both dynasore and cisplatin treatment significantly decreased the EdU positive rate in OS cells (17.80 ± 1.56% in dynasore group and 3.46 ± 0.45% in cisplatin group vs. 50.51 ± 1.42% in control group in MNNG/HOS, *p* < 0.01; 19.27 ± 0.95% in dynasore group and 6.75 ± 1.14% in cisplatin group vs. 33.37 ± 0.85% in control group in MG-63, *p* *<* 0.01). Consistent with our previous results, the EdU positive rate of dynasore and cisplatin combination group (MNNG/HOS: 0.77 ± 0.08%, *p* *<* 0.01; MG-63: 1.59 ± 0.14%, *p* *<* 0.01) were significantly lower than cisplatin group. In addition, cloning formation assay demonstrated similar results (Fig. 2e, f). However, with the shadow of the powerful anti-colony formation effect of cisplatin in MG-63, dynasore decreased the relative colony formation rate with no statistical evidence (0 ± 0% vs. 1 ± 0.71%, *p* *=* 0.158).

Thus, these results indicated that dynasore significantly suppressed viability, proliferation, and colony formation of OS cells, and has synergy effect with cisplatin.

### Dynasore induces G0/G1 arrest but not cell apoptosis of OS cells

**Fig. 3:**
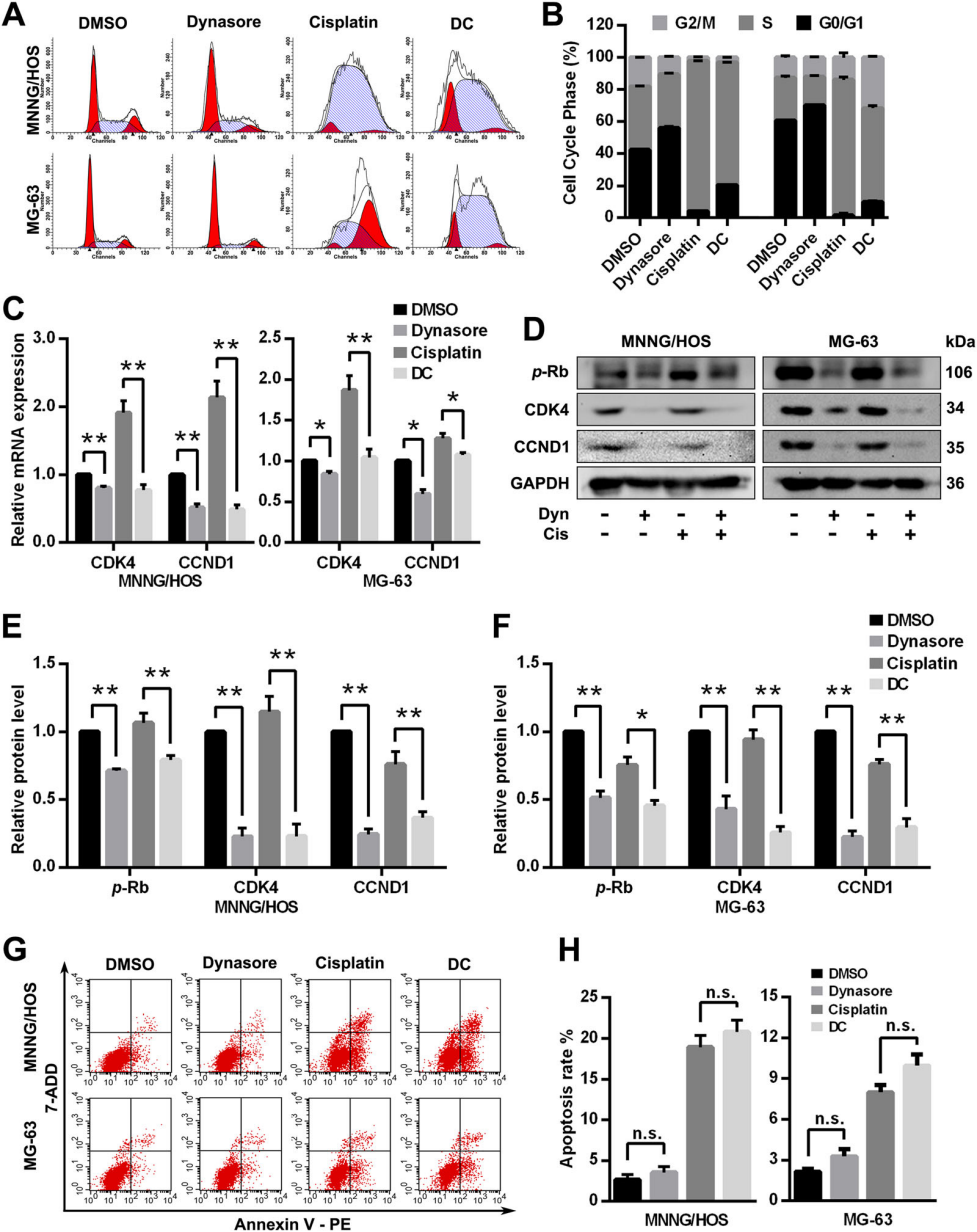
Dynasore induces G0/G1 cell cycle arrest but not cell apoptosis of OS cells. **a** The representative data of PI staining and folw cytometric analysis of OS cells treated with dynasore and/or cisplatin. **b** Histograms of cell cycle distribution of MNNG/HOS (left) and MG-63 (right) show that dynasore significantly increases the percentages of cells in G0/G1 phase. **c** The relative mRNA expression of CDK4 and CCND1 in OS cells. **d** Representative western blot analysis for *p*-Rb, CDK4, and CCND1 in OS cells. Relative protein levels of *p*-Rb, CDK4, and CCND1 in MNNG/HOS (**e**) and MG-63 (**f**) cells are shown as histograms. **g** The representative data of cell apoptosis. **h** Histograms of cell apoptosis rates of OS cells. *p < 0.05 and **p < 0.01 vs. DMSO group. n.s., not significant

The cell cycle distribution and apoptosis of MNNG/HOS and MG-63 were evaluated using flow cytometry after treatment with dynasore and cisplatin alone or in combination. As revealed in Fig. 3a, b, dynasore exposures led to accumulation in G0/G1 phase both in MNNG/HOS (56.04 ± 1.08% vs. 42.36 ± 0.25%, *p* < 0.01) and MG-63 cells (70.30 ± 0.50% vs. 60.74 ± 0.11%, *p* < 0.01), while cisplatin accumulated cells in S phase (93.62 ± 0.38% vs. 39.50 ± 0.44% in MNNG/HOS; 84.71 ± 2.60% vs. 26.63 ± 1.67% in MG-63, *p* < 0.01). Surprisingly, compared with cisplatin group, dynasore combined with cisplatin significantly increased the population of OS cells in G0/G1 phase (20.39 ± 0.21% vs. 4.09 ± 0.05% in MNNG/HOS and 9.87 ± 1.27% vs. 1.52 ± 1.92% in MG-63). To further elucidate the mechanisms, the transcription and expression of proteins essential for cell cycle G1/S transition were analyzed via real-time PCR and western blot, respectively. As shown in Fig. 3c–f, dynasore down-regulated both mRNA and protein levels of CCND1 and CDK4. Moreover, the phosphorylation of Rb, a protein that sequesters E2F to suppress transcription activation, was significantly inhibited by dynasore in both MNNG/HOS and MG-63 cells. Compared with cisplatin treatment, the expression of *p*-Rb, CCND1, and CDK4 were also down-regulated when dynasore was added.

Furthermore, cell apoptosis was detected in MNNG/HOS and MG-63 (Fig. 3g, h). Astonishingly, although dynasore treatment for 48 h mildly increased apoptosis of OS cells, statistically significant differences were found neither between dynasore group and control group (*p* = 0.390 and *p* = 0.116) nor between DC group and cisplatin group (*p* = 0.386 and *p* = 0.121).

Therefore, these results suggests that dynasore induces G0/G1 arrest through inhibiting the expression of *p*-Rb, CCND1, and CDK4, while not affects apoptosis of OS cells in vitro.

### Dynasore suppresses OS cell migration and invasion

**Fig. 4:**
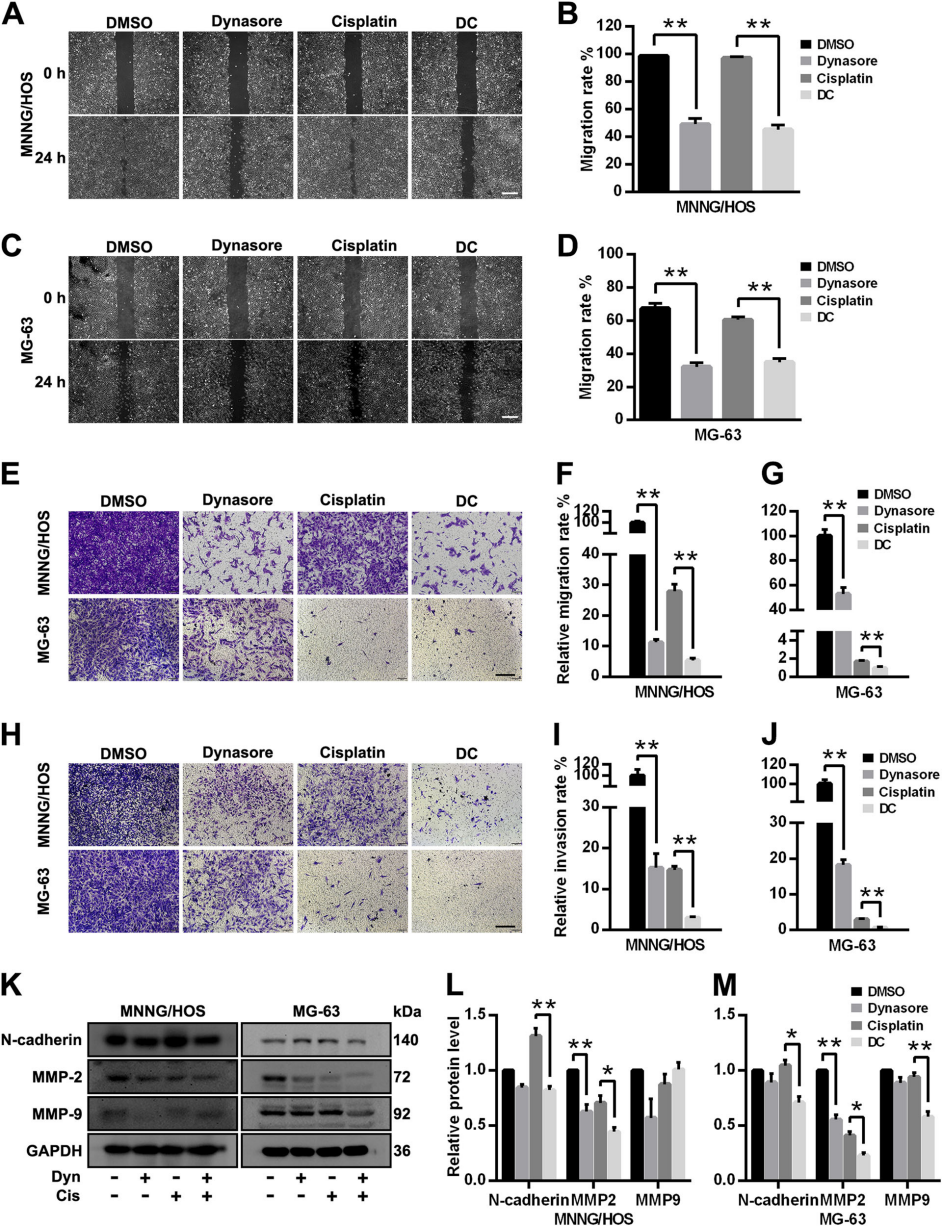
Dynasore suppresses the migration and invasion capacity of OS cells. The representative data and histograms of wound healing assays of MNNG/HOS (**a**, **b**) and MG-63 (**c**, **d**) (Scare bar: 400 μm). Transwell assays show that dynasore and cisplatin alone or in combination both can inhibit the migration (without Matrigel, **e**–**g**) and invasion capacity (with Matrigel, **h**–**j**) of OS cells (Scare bar: 200 μm). **k** Western blot analysis of N-cadherin, MMP-2 and MMP-9 in OS cells. The relative protein expressions of N-cadherin, MMP-2 and MMP-9 in MNNG/HOS (**l**) and MG-63 (**m**) cells are shown as histograms. *p < 0.05 and **p < 0.01 vs. DMSO group

The capacity of migration and invasion play a crucial role in OS distant metastasis and local invasion. As shown in Fig. 4a–d, the wound healing assay revealed that dynasore significantly delayed the lessening of the scratch area in both OS cells, and migration rates were significantly decreased in DC group when compared with cisplatin group. Consistent with wound healing assay, the transwell migration (Fig. 4e–g) and invasion assays (Fig. 4h–j) exerted the same pattern of effects of dynasore and cisplatin, indicating that dynasore exposure inhibited cell migration and invasion capacity and enhanced the inhibitory effect of cisplatin in OS cells. Furthermore, we examined cell migration an invasion relevant proteins (N-cadherin, MMP-2, and MMP-9) after treatment with dynasore and cisplatin alone or in combination. As exhibited in Fig. 4k–m, both dynasore and cisplatin alone suppressed the expression of MMP-2 significantly in MNNG/HOS and MG-63 cells, and the effects are more prominent in combination group. However, dynasore combined with cisplatin decreased N-cadherin expression when compared with cisplatin in both cells, while no effects on N-cadherin were observed when dynasore was used alone.

### Dynasore inhibits OS tumorigenesis without inducing nephrotoxicity and hepatotoxicity in vivo

**Fig. 5:**
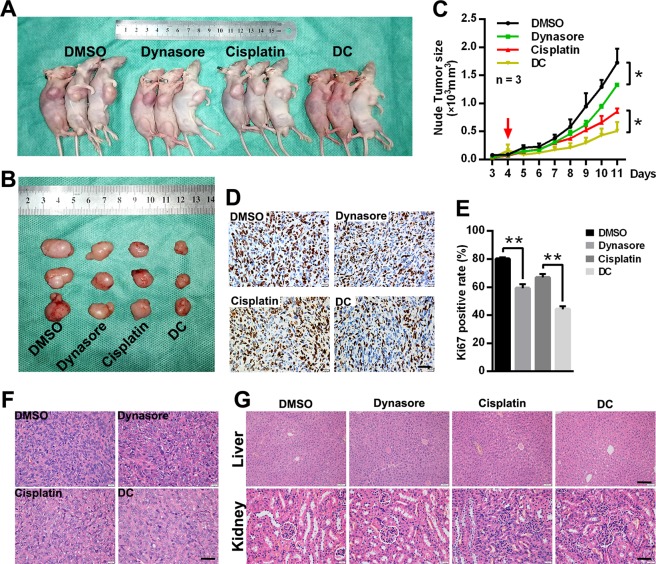
Effects of dynasore and cisplatin in OS xenograft mouse model. **a**, **b** The representative mice and tumors of the xenograft mouse modes in DMSO, dynasore (10 mg/kg), cisplatin (2 mg/kg) and combination-treated groups. **c** Tumor volumes were measured every day since day 3 after model establishment (day 0), and drugs administration were initiated at day 4 (red arrow). **d**, **e** Xenograft tumor tissues were subjected to IHC staining by using Ki67 antibody (Scare bar: 40 μm). Tumor samples (**f**) (Scare bar: 40 μm), liver (Scare bar: 100 μm) and kidney (Scare bar: 40 μm) samples (**g**) of four xenograft mice groups were under H&E staining. *p < 0.05 and **p < 0.01 vs. DMSO group

In order to determine the potential value of dynasore in OS treatment, a xenograft mouse model was established and the volumes of tumors were measured every day. The administration of dynasore resulted in significantly decreased in tumor volume (Fig. 5a–c, *p* *<* 0.05). Furthermore, DC group gained the greatest suppression effect in OS growth (Fig. 5a–c, *p* *<* 0.05), while the mean tumor volume in cisplatin group were in between dynasore group and DC group. As revealed in in vitro assays, dynasore highlights an anti-proliferation effect when treated alone or combined with cisplatin in OS cells. Therefore, the expressions of Ki67 were detected in xenografts by using IHC (Fig. 5d, e). Dynasore and cisplatin alone both significantly reduced Ki67 expression in vivo. In addition, the average Ki67 positive rate was further decreased when dynasore and cisplatin were administrated together. As shown in Fig. 5f, the OS cell densities in xenografts were much lower when treated with dynasore, while cisplatin alone caused mainly cell death and fatty degeneration. Besides, tumors in DC group had features of dynasore and cisplatin groups, and achieved a better effect of growth suppression in vivo.

Furthermore, the histomorphology of liver and kidneys of xenografts mice was analysis by H&E staining (Fig. 5g). Surprisingly, no obvious evidences showed that dynasore induced nephrotoxicity or hepatotoxicity in the present study. However, in cisplatin treated mice, edematous glomeruli and swollen and degenerated renal tubular epithelial cells were observed.

Taken together, dynasore exerted anti-tumorigenesis effect mainly by inducing proliferation repression on OS without causing or nephrotoxicity or hepatotoxicity.

### P38 MAPK signaling pathway is involved in dynasore induced anti-osteosarcoma effects

**Fig. 6:**
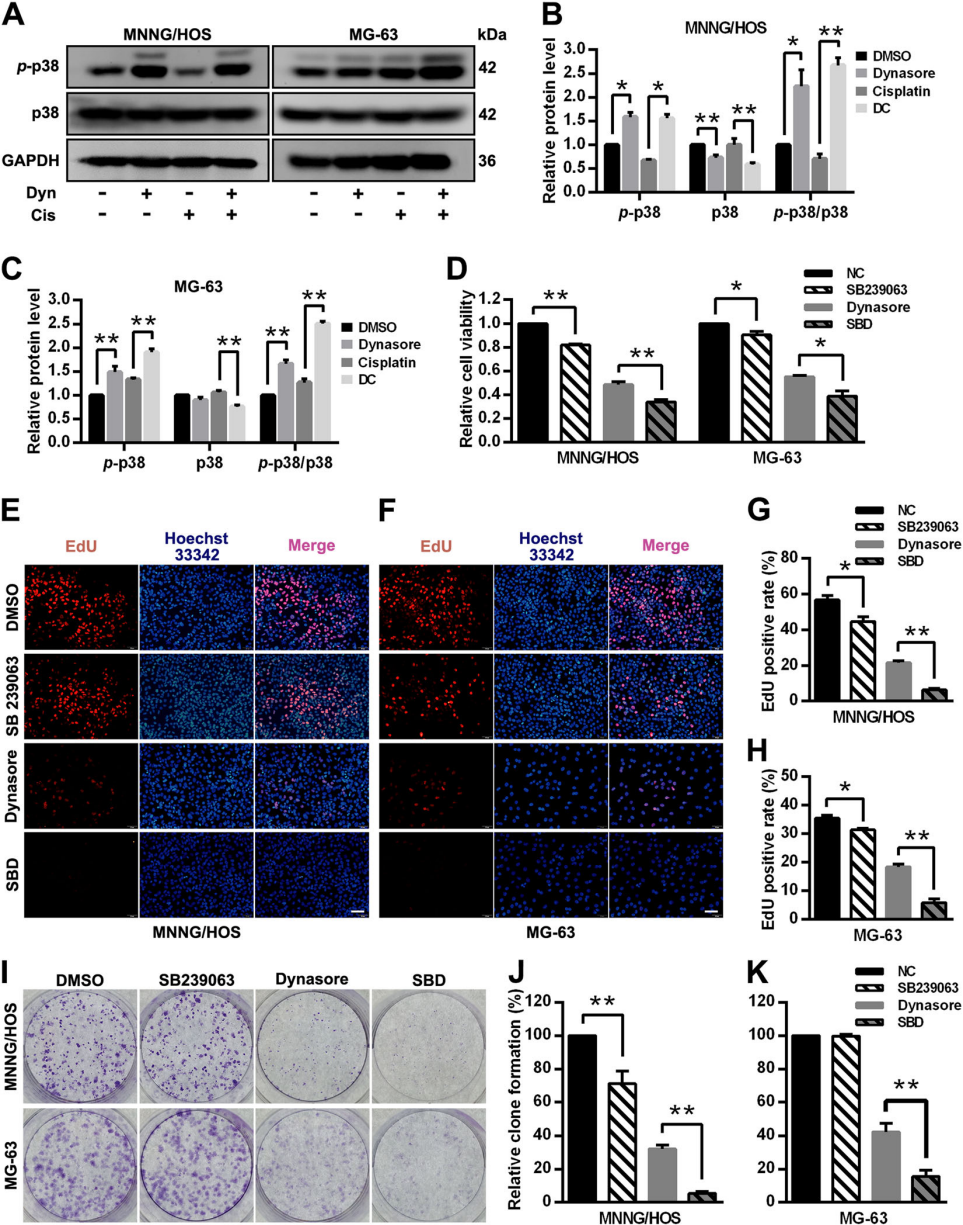
P38 MAPK signaling pathway serves as a negative regulator in dynasore induced proliferative inhibition. **a** Western blot analysis of *p*-p38 and p38 of OS cells after treated with dynasore and/or cisplatin. The relative protein expression of p-p38 and p38 in MNNG/HOS (**b**) and MG-63 (**c**) were shown as histogram. **d** The cell viability of OS cells after treated with SB239063 (10 μM) followed by dynasore exposure. After suppression of p38 MAPK pathway by SB239063, cell proliferations were analyzed by EdU staining (MNNG/HOS (**e**, **g**) and MG-63 cells (**f**, **h**)) and cloning formation assay (I-K). *p < 0.05 and **p < 0.01 vs. DMSO group. SBD, co-treated with 50 μM dynasore and 10 μM SB239063 as described in “Materials and methods” section. Scale bar: 50 μm

**Fig. 7:**
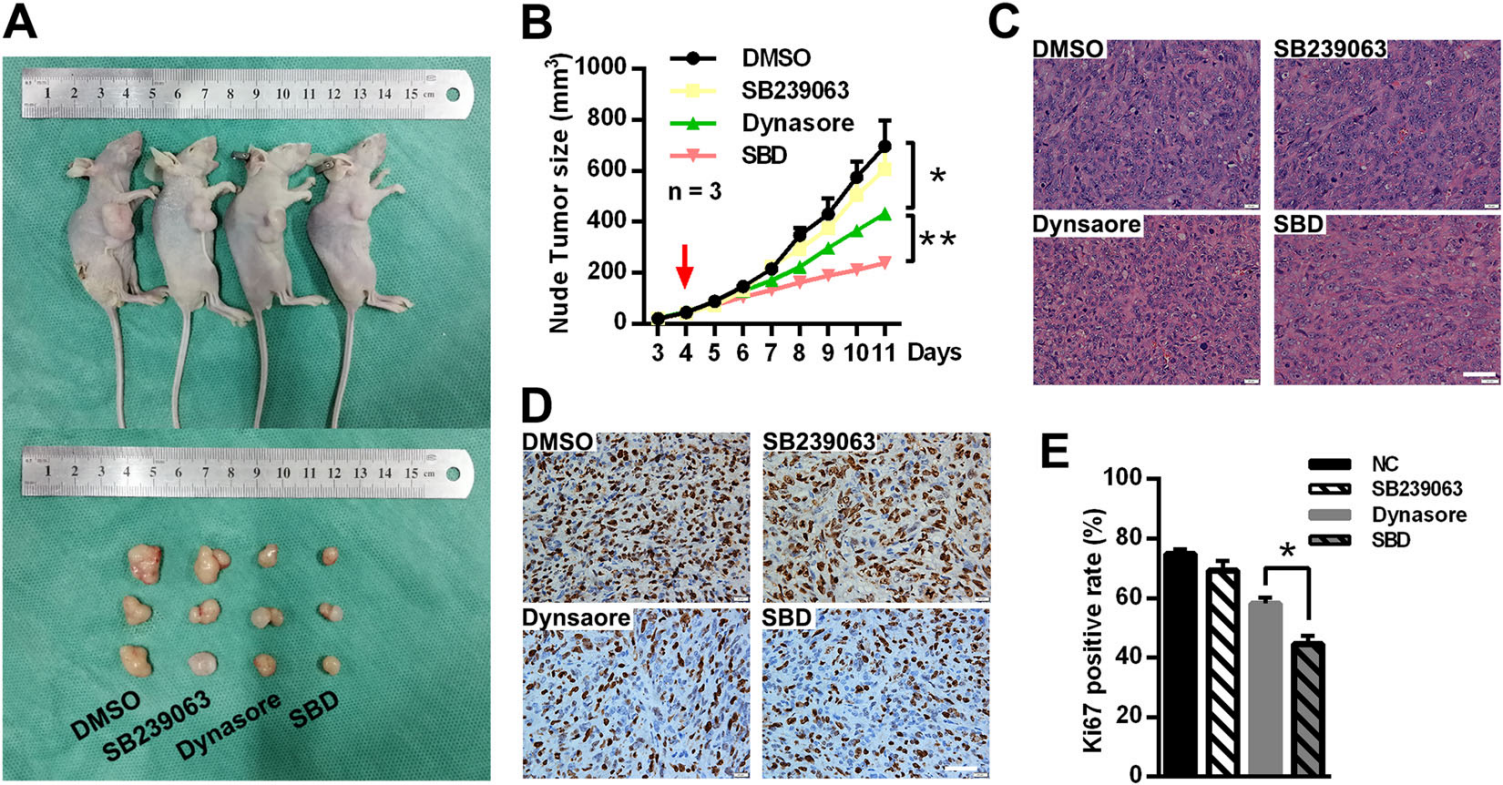
Effects of p38 MAPK pathway inhibitor SB239063 in dynasore induced anti-OS in vivo. **a** The representative mouse and tumors of the xenograft mouse modes in DMSO, SB239063 (10 mg/kg), dynasore (10 mg/kg) and combination-treated groups. **b** Tumor volumes were measured every day since day 3 after model establishment (day 0), and drugs administration were initiated at day 4 (red arrow). **c** Tumor samples of four xenograft mice groups were under H&E staining (Scare bar: 40 μm). **d**, **e** Xenograft tumor tissues were subjected to IHC staining by using Ki67 antibody (Scare bar: 40 μm). *p < 0.05 and **p < 0.01 vs. DMSO group

Next, western blot was conducted to further explore the signaling pathways involved in dynasore induced anti-proliferation effect. Dynasore significantly increased the phosphorylation levels of p38 (Fig. 6a–c). Moreover, as expected, *p*-p38 level was up-regulated in DC group compared with cisplatin group. These results indicated that p38 MAPK signaling pathway was involved in dynasore induce anti-osteosarcoma effects. However, when SB239063, a specific inhibitor of p38 MAPK, was applied to inhibit the activation of this pathway, dynasore enhanced its anti-proliferation effect dramatically. The cell ability suppression capacities of dynasore in MNNG/HOS and MG-63 were more prominent after pre-treated with SB239063 (Fig. 6d). EdU staining and colony formation assay demonstrated the same pattern of effects of dynasore when SB239063 was used (Fig. 6e–k). In in vivo assay, the tumor growth was not affected by SB239063. However, the tumor volumes in the group treated with dynasore and SB239063 were significantly smaller than those in dynasore group (Fig. 7a, b). By H&E staining, we found that the cell density in xenografts of SB239063 and dynasore combination group (SBD group) was much sparser than that in dynasore group (Fig. 7c). In addition, the average ki67 positive rate in SBD group was significantly lower than that in dynasore group (44.62 ± 2.73% vs. 58.23 ± 3.33%, *p* < 0.05, Fig. 7d, e). Taken together, p38 MAPK pathway might serves as a negative regulator in dynasore induced proliferation inhibition. However, the phosphorylation of ERK1/2, Akt and JNK did not have significant differences between control and dynasore treated group (Supplemental Fig. 4), indicating that MAPK ERK1/2, PI3K-Akt, and SAPK/JNK pathways might not involve in repression of OS proliferation induced by dynasore.

### Dynasore inhibits cell proliferation via STAT3 pathway

**Fig. 8:**
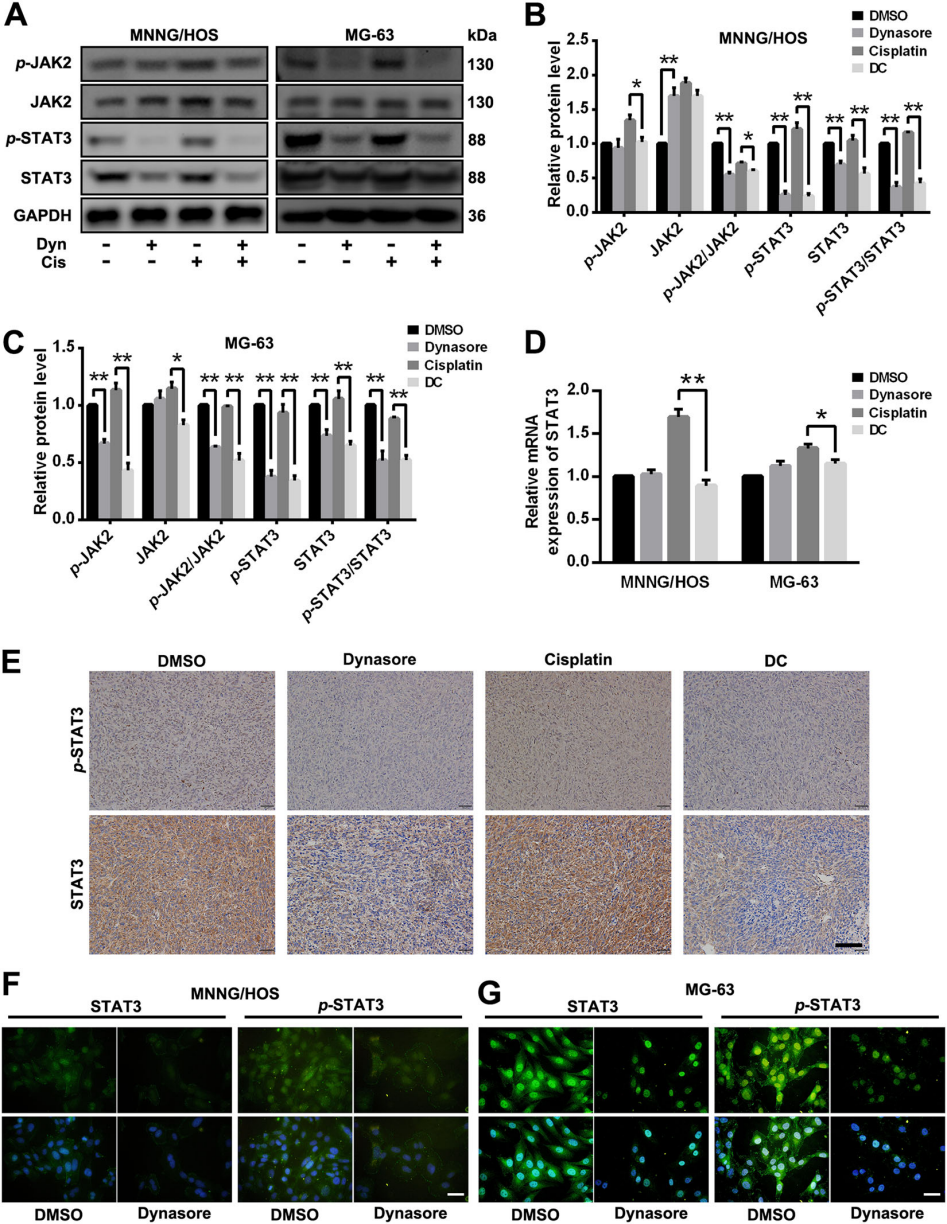
Dynasore inhibit the activation of STAT3 signaling pathway. **a** Western blot analysis of *p*-JAK2, JAK2, *p*-STAT3 and STAT3 of OS cells after treated with dynasore and/or cisplatin. The relative protein expression of *p*-JAK2, JAK2, *p*-STAT3, and STAT3 in MNNG/HOS (**b**) and MG-63 (**c**) were shown as histogram. **d** The mRNA expression of STAT3 in MNNG/HOS and MG-63 cells after dynasore and/or cisplatin exposure for 48 h. **e** The *p*-STAT3 and STAT3 expression in xenograft tumor tissues were detected by using IHC staining (Scale bar: 100 μm). The *p*-STAT3 and STAT3 expression were detected by using immunohistochemistry assay in MNNG/HOS (**f**) and MG-63 cells (**g**) (Scale bar: 20 μm). *p < 0.05 and **p < 0.01 vs. DMSO group

**Fig. 9:**
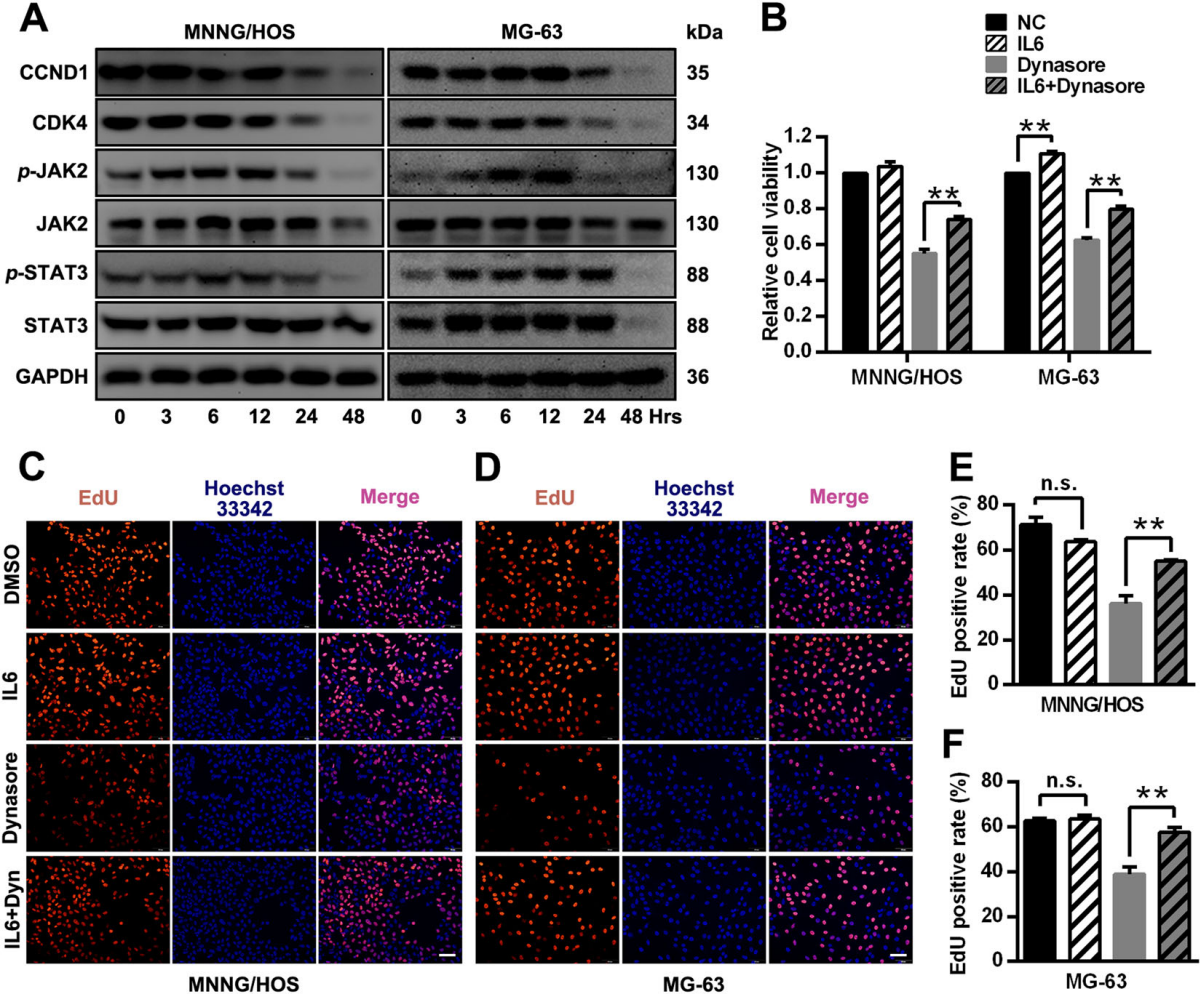
Dynasore exert anti-OS effects partially by STAT3 signaling pathway. **a** The expression of CCND1, CDK4, *p*-JAK2, JAK2, *p*-STAT3, and STAT3 were detected by western blot after treated with dynasore for 0, 3, 6, 12, 24, and 48 h. **b** The cell viability of OS cells after treated with IL-6 (50 ng/ml) followed by dynasore exposure. After activation of STAT3 pathway by IL-6, cell proliferations were analyzed by EdU staining (MNNG/HOS (**c** and **e**) and MG-63 cells (**d** and **f**)). *p < 0.05 and **p < 0.01 vs. DMSO group. Hrs, hours. Scale bar: 50 μm

In the previous results, we found p38 MAPK, which acted as a negative regulator, was involved in the effects of dynasore on OS. Here, we further studied the role of JAK2/SAT3 pathway in dynasore-induced inhibition of OS proliferation. The phosphorylations of JAK2 were significantly suppressed by dynasore in both MNNG/HOS and MG-63 cells (Fig. 8a–c). Surprisingly, the expression of STAT3 and *p*-STAT3 were both significantly down-regulated in dynasore group, and the *p*-STAT3 levels were indeed repressed by dynasore when standardized to total STAT3 expression (Fig. 8a–c). Moreover, the expressions of these proteins were reduced in DC group when compared with cisplatin group. Furthermore, by using real-time PCR, no significant difference of mRNA expression of STAT3 was found in dynasore group and control group. However, up-regulated STAT3 transcription levels, which was reversed by dynasore in DC group, was detected after cisplatin intervention (Fig. 8d). Consistent with in vitro detection, the *p*-STAT3 and STAT3 expression were decreased in xenografts OS tumors exposed to dynasore (Fig. 8e). To intuitively observe the effects of dynasore on subcellar location of STAT3 and *p*-STAT3, immunofluorescence experiments were performed. As exhibited in Fig. 8f, g, the fluorescence intensities of STAT3 and *p*-STAT3 were decreased both in nucleus and cytoplasm in dynasore treated group. In order to investigate the relationship between cell cycle arrest and JAK2/STAT3 pathway repression, the expressions of CCDN1, CDK4 and phosphorylation of JAK2 and STAT3 were evaluated at 0, 3, 6, 12, 24, 48 h after dynasore treatment. As expected (Fig. 9a), the CCND1, CDK4, *p*-JAK2, and *p*-STAT3 levels started decreasing at 24 h and were dramatically reduced at 48 h, simultaneously.

Furthermore, after activated by inflammatory cytokine Interleukin-6 (IL-6), a cytokine that was identified as a well-established STAT3 signaling pathway activator^22^, the effects of dynasore on cell viability and proliferation of OS cells were determined by CCK-8 assay and EdU staining. The inhibitory effect of dynasore on cell viability (Fig. 9b) and proliferation (Fig. 9c–f) was almost abrogated by IL-6 stimuli, indicating that dynasore inhibited OS cell proliferation via deactivating STAT3 pathway.

## Discussion

The prognosis of patients with OS has been improved with the implementation of chemotherapy in the 1970s^2^. However, despite the unremitting efforts of researchers in the last four decades, the survival rate still remains unsatisfactory, especially in patients with systemic metastasis or local relapse^2,23–25^. Drugs that widely used in OS chemotherapy generally develop side effects and cause injuries in normal tissues of cancer patients. Worse still, about 35–45% patients turn out to be unresponsive or resistant to these drugs, which leads to treatment failure^2,26^. In the present study, we demonstrated the anti-OS potential of dynasore for the first time in vitro and in vivo model. By combining dynasore and cisplatin, we found that dynasore enhanced the anti-tumor effect of cisplatin.

Cell cycle is a sequence of highly regulated events that allows a cell to grow, duplicate its genetic material, and divide into two daughter cells^27^. The homeostasis of the cell cycle regulatory mechanisms is deranged in cancer cells, and consequently, leading to unscheduled malignant proliferation, which is considered as one of the proverbial features of malignancies^28^. In G0/G1 phase, the activity of E2F, which is critical in gene transcription, was repressed by hypophosphorylated pRb. Once triggered by G1-S cell cycle transitive signaling, the increasingly synthesized CCND binds to cyclin-dependent kinases (CDK4 or CDK6). Then, the CCND-CDK4/6 complexes phosphorylate pRb to release the pRb from E2F-DP complexes and initiate the activation of transcription^27,29,30^. We found that dynasore suppressed cell proliferation and induced G0/G1 phase arrest of OS cell lines. Furthermore, dynasore reduced the expression of pRb, CCND1, and CDK4. These results indicate that dynasore somehow inhibited CCND1 and CDK4 expression so that CCND1-CDK4 complexes decreased, which leads to hypophosphorylation of pRb and block G1-S transition. Since identified, dynasore was widely used in the researches of endocytosis and macropinocytosis as a dynamin inhibitor^11,12,31–33^. In the latest reports, dynasore was found to be able to repress cell proliferation, migration of lung cancer cell lines in vitro^18,19^, and, consist with our results, dynasore augmented the anti-cancer effects of cisplatin^18^. In the present study, no sufficient evidences were found in dynasore induced apoptosis of OS cells. However, dynasore induced apoptosis and mitochondrial dysfunction in lung cancer cells. Moreover, dynasore induced S phase cell cycle arrest in HCC827 and H1650 cells, while did not affect the cell cycle distribution of A549 cells^19^. These differences indicate the diverse roles of dynasore in different cancer cells.

Abnormally elevated cell migration and invasion gives OS cells the intensive ability of systemic metastasis and locally aggression, which is responsible for poor prognosis and death^2^. N-cadherin is a transmembrane protein which mediates intercellular adhesion and involves in transendothelial migration. In Epithelial–Mesenchymal Transition (EMT) process, the expression of N-cadherin is increased and balanced by E-cadherin down-regulation. The transitioned cells acquire the affinity to mesenchymal cells and enhance the expression of proteases such as matrix metalloproteinases (MMPs), and, finally facilitate the capacity of migration and invasion^34,35^. The MMPs are a family of zinc-containing endopeptidases which are capable of degrading all kinds of extracellular matrix (ECM) protein, and play important roles in physiological and pathological processes^36,37^. Among these MMPs, MMP-2, and MMP-9 were confirmed to be crucial in cancer metastasis and aggression for their ability of remodeling basement membrane via degrading Type IV collagen^38,39^. Chung et al. showed that elevated MMP-2 activity stimulated by TGF-β1 was partially impaired when pretreated with dynasore^40^. Wang et al found dynasore inhibited the migration of lung cancer cells and down-regulated active MMP-9 level^19^. Consistently, our results showed that dynasore treatment resulted in suppressed cell migration and invasion, and reduced MMP-2 protein level of OS cells. However, western blot found no evidences of dynasore mediated MMP-9 or N-cadherin down-regulation. Therefore, dynasore inhibited the migration and invasion of OS mainly through down-regulation of MMP-2, but not MMP-9 or N-cadherin.

STAT3 is a member of STATs (Signal Transducers and Activators of Transcriptions) family and acts as signal messagers and transcription factors. After activated by cytokines or growth factors, STAT3s are phosphorylated to form STAT3 dimer and travels from plasma membrane to nucleus, and then initiates the transcription of downstream target genes such as CCND1, CCNB, cdc2, surviving, Bcl-2, Mcl-1, MMP-1, and MMP-2^41–44^. In cancer cells, constitutive activation of STAT3 breaks the strict control mechanism of downstream gene transcription and involves in tumor growth, survival, invasion, metastasis, and angiogenesis^45,46^. Literatures have shown that over-expression and aberrant activation of STAT3 contributes to the tumorigenesis, progression, and poor prognosis of several cancers including OS^47–50^. Besides, growing concentrations have focused on novel drugs investigation by treating STAT3 as a therapy target^51–53^. Our study showed that dynasore treatment inhibited cell proliferation, migration, invasion and tumorigenesis of OS, decreased the expression of *p*-STAT3 in vitro and in xenograft mouse model. At the meantime, total STAT3 protein expression was found suppressed by dynasore in vitro and in vivo. By using real-time PCR, we found that the mRNA expression of STAT3 in MNNG/HOS and MG-63 was not affected by dynasore, which indicating that the protein expression of STAT3 was inhibited at post-transcriptional level. The post-transcriptional modification of STAT3 includes tyrosine phosphorylation, serine phosphorylation, acetylation, methylation, and oxidation^54^. STAT3 deacetylation can be activated by NAD-dependent silent information regulator protein (SIRT) 1, and results STAT3 destabilization and degradation^54–56^. By treating with dynasore, the endocytosis of cells is repressed, which might block the cellular nutrients transportation and, consequently, activate regulator proteins like SIRT1 to initiate STAT3 deacetylation. Literatures showed that most STAT3 inhibitors^53,57,58^ functioned by repressing the activation of STAT3. Our study indicated that dynasore served as a STAT3 pathway inhibitor in OS, which supplement the list of inhibitors^59,60^ suppressing both expression and phosphorylation of STAT3. In addition, the phosphorylation of JAK2, which can activate STAT family, and downstream target genes (CCND1, MMP-2) of STAT3, was also down-regulated in dynasore exposed group. IL-6 is the most well known activator of STAT3 pathway^61,62^. In this study, we activated the STAT3 pathway by using IL-6 and found that the anti-proliferation effect of dynasore on OS cells was dramatically attenuated (both in CCK-8 assay and EdU staining assay). These evidences suggest that dynasore exerts anti-tumor effect, or at least anti-proliferation effect, in OS via STAT3 signaling pathway.

Previous studies have indicated that p38 MAPK, MAPK-ERK, PI3K-Akt, and SAPK/JNK signaling pathways are involved in cell proliferation^20,63–66^. In our study, we found that dynasore did not affect the expression and activation of ERK1/2, Akt or SAPK/JNK, indicating that MAPK-ERK and PI3K-Akt and SAPK/JNK signaling pathways might not involve in dynasore induced anti-OS effect. The role of p38 MAPK pathway in proliferation was ambiguous^66^. Most studies suggested that p38 activation promoted cell proliferation^64,67,68^, while another reports showed opposite results^65^. Here, we demonstrated that the phosphorylation level of p38 was elevated in dynasore treated OS cells. Interestingly, by using SB239063, a p38-specific inhibitor, the anti-proliferation effect of dynasore was enhanced. Collectively, we hypothesize that when dynasore significantly inhibited cell proliferation, p38 MAPK pathway was activated as a negative regulatory mechanism to lessen the anti-tumor effect of dynasore on OS cells. Therefore, if possible, p38 MAPK inhibitor should be used as an adjuvant of dynasore in the treatment of OS in the future.

In summary, to our knowledge, this is the first research that studies the anti-tumor effect of dynasore on OS. Based on our finding, we exhibited that dynasore possessed anti-OS ability in vitro and in vivo. Besides, dynasore significantly enhanced the anti-tumor effects of cisplatin without inducing nephrotoxicity and hepatotoxicity. Mechanistically, we found that dynasore exerted anti-tumor effects, or at least anti-proliferation effect, in OS via STAT3 signaling pathway. Collectively, our study reveals the anti-tumor effects of dynasore on OS, which may supplement the candidate chemotherapy drug list, and reduce cisplatin-mediated side effects by decreasing dosage via drug combination.

## Supplementary information

Supplemental Figure 1

Supplemental Figure 2

Supplemental Figure 3

Supplemental Figure 4
