# Supplementary figures and images for: Dynasore suppresses cell proliferation, migration, and invasion and enhances the antitumor capacity of cisplatin via STAT3 pathway in osteosarcoma
Source: Cell Death Dis. 2019 Sep 18;10(10):687. doi: 10.1038/s41419-019-1917-2 (PMC6751204; doi:10.1038/s41419-019-1917-2)

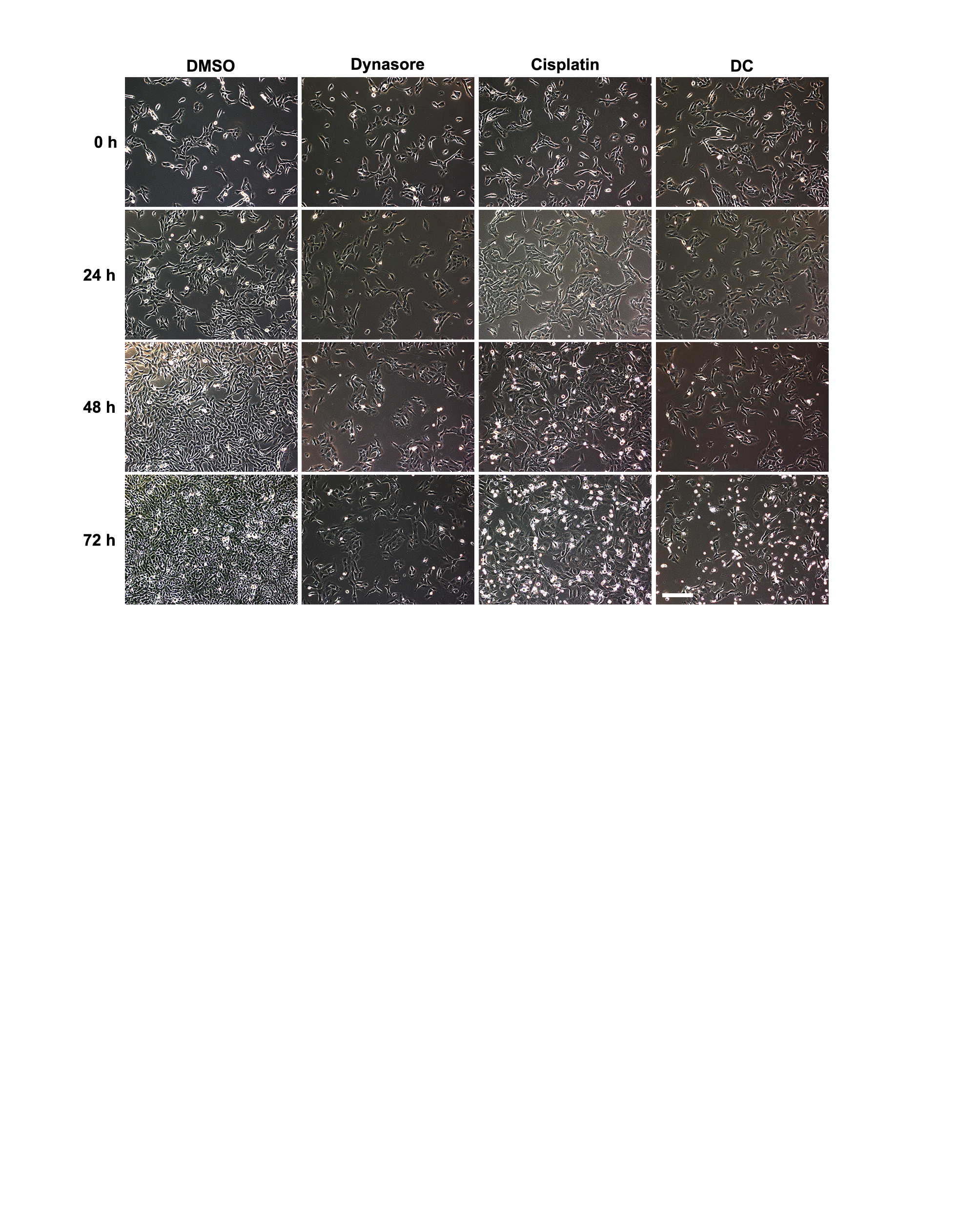

Supplement: Supplementary file 1 — Supplemental Figure 1 [file 41419_2019_1917_MOESM1_ESM.tif]

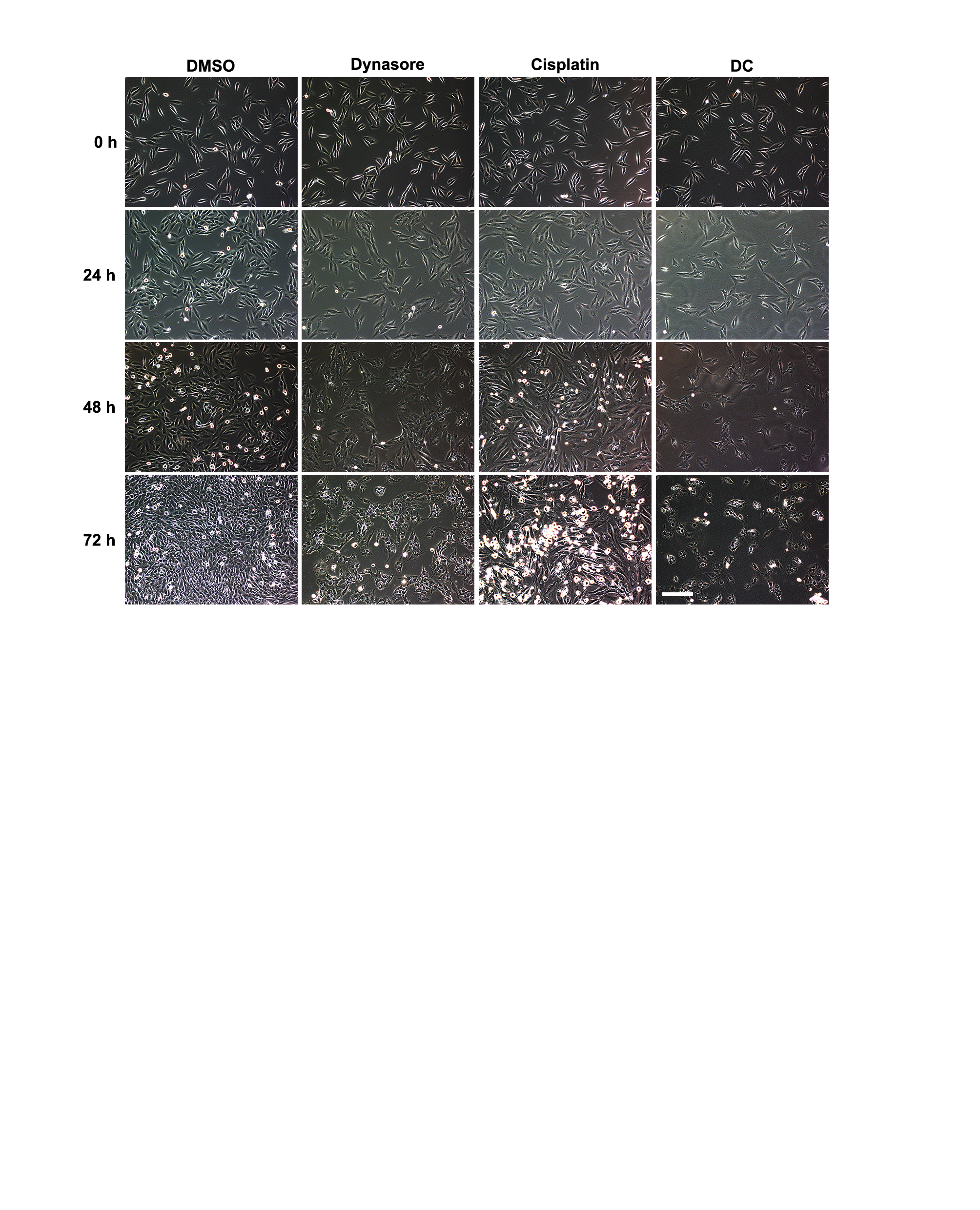

Supplement: Supplementary file 2 — Supplemental Figure 2 [file 41419_2019_1917_MOESM2_ESM.tif]

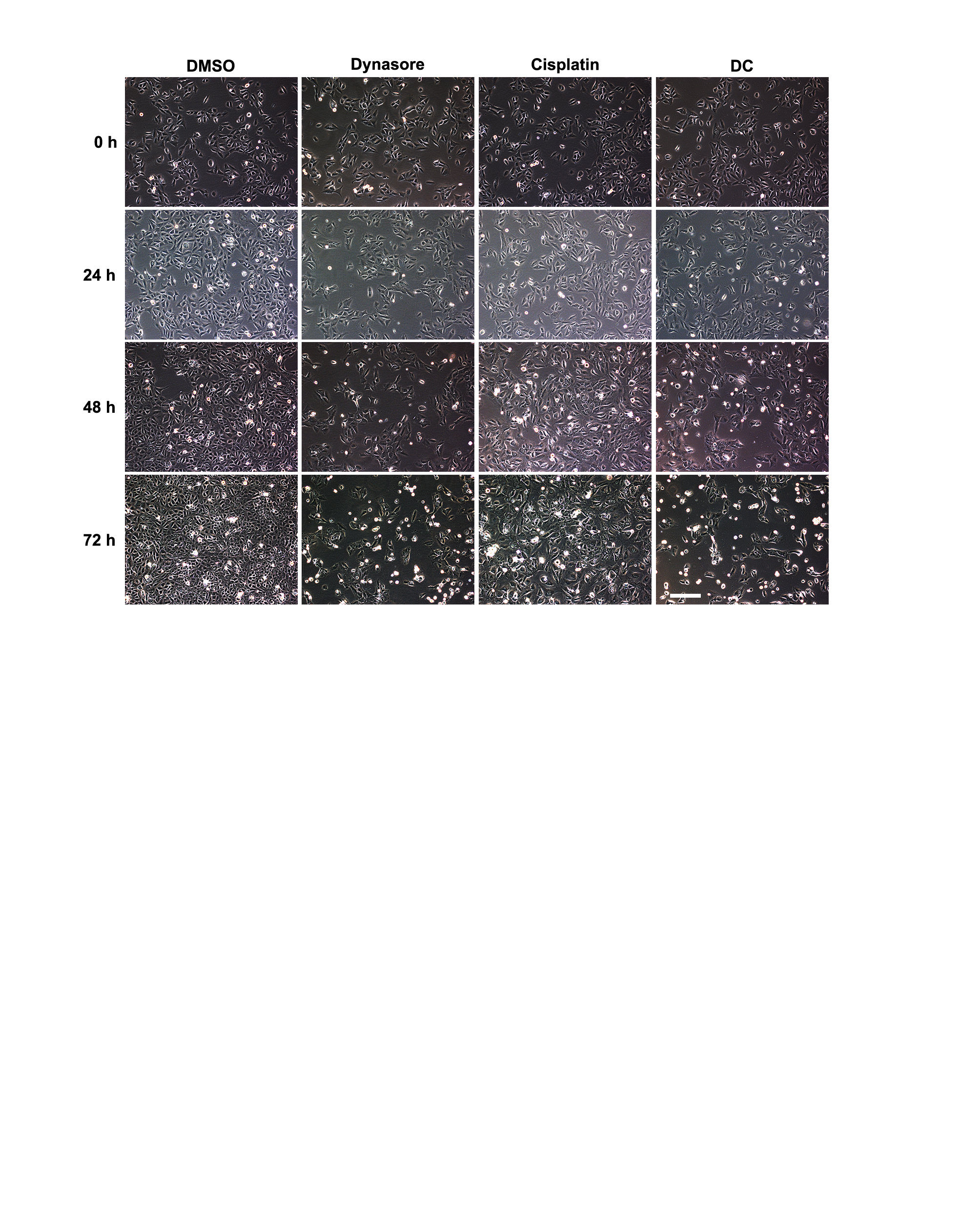

Supplement: Supplementary file 3 — Supplemental Figure 3 [file 41419_2019_1917_MOESM3_ESM.tif]

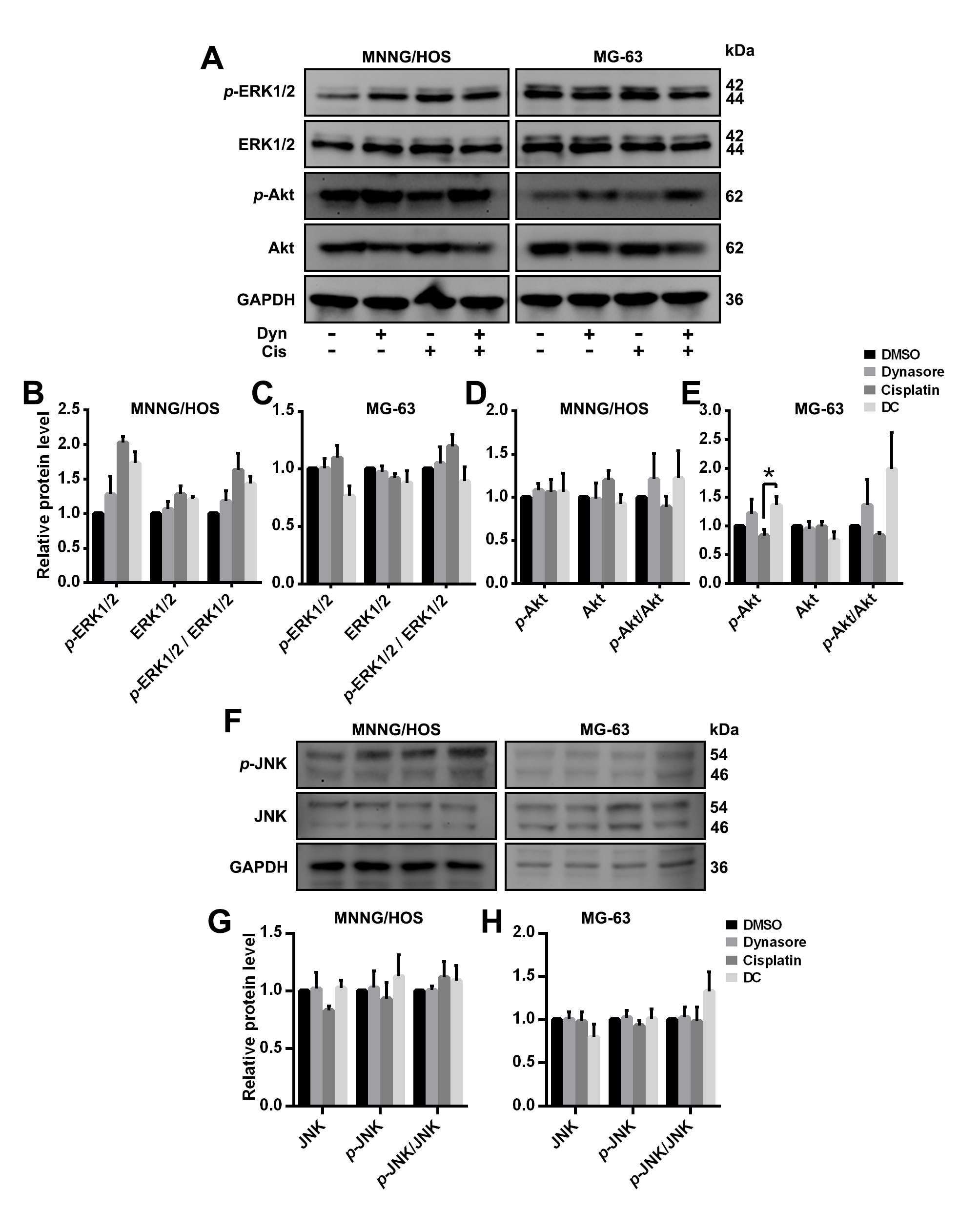

Supplement: Supplementary file 4 — Supplemental Figure 4 [file 41419_2019_1917_MOESM4_ESM.tif]
